# Semaglutide in Heart Failure With Preserved Ejection Fraction: Emerging Evidence and Clinical Implications

**DOI:** 10.7759/cureus.87605

**Published:** 2025-07-09

**Authors:** FNU Arty, Devarashetty Shreya, Aleeza Chaudhry, Sarkar Sohini

**Affiliations:** 1 Internal Medicine, Monmouth Medical Center, Long Branch, USA

**Keywords:** clinical trials, glp-1 receptor agonist, heart failure management, heart failure with preserved ejection fraction, hfpef, obesity, ozempic, semaglutide

## Abstract

Heart failure with preserved ejection fraction (HFpEF) poses a growing public health challenge, characterized by limited therapeutic options and a high burden of comorbid obesity and type 2 diabetes (T2D). Semaglutide, a glucagon-like peptide-1 receptor agonist (GLP-1 RA), has shown promise in addressing these comorbidities, offering potential benefits in obesity-related HFpEF. We analyzed key clinical trials, including SUSTAIN 6, PIONEER 6, STEP HFpEF, STEP HFpEF DM, and supportive studies on glucagon-like peptide receptor agonists (GLP-1 RAs) and sodium-glucose cotransporter 2 (SGLT2) inhibitors, alongside mechanistic insights into semaglutide’s metabolic, anti-inflammatory, and decongestive effects. Data were drawn from randomized controlled trials, cohort studies, and pathophysiological reviews. Semaglutide significantly improves HFpEF outcomes in obese patients, with STEP HFpEF (n = 529) and STEP HFpEF DM (n = 616) demonstrating Kansas City Cardiomyopathy Questionnaire Clinical Summary Score increases of 16.6 and 13.7 points (vs. 8.7 and 6.4 with placebo, P < 0.001), weight reductions of 13.3% and 9.8% (P < 0.001), and 43.5% C-reactive protein reductions (P < 0.001), all of which are clinically relevant in HFpEF management. SUSTAIN 6 and PIONEER 6 highlight cardiovascular safety in T2D, reducing stroke and mortality, while combination with SGLT2 inhibitors lowers HF exacerbations (HR 0.62). However, these findings are primarily derived from trials enrolling patients with obesity and relatively well-preserved renal and cardiac function, limiting applicability to the broader, heterogeneous HFpEF population. The included trials differed substantially in design: STEP trials focused on short-term, functional outcomes over 52 weeks; SUSTAIN 6 and PIONEER 6 assessed cardiovascular safety in T2D populations with varied HF status. Follow-up durations, primary endpoints, and inclusion criteria also varied across studies. This heterogeneity complicates direct comparison and limits the ability to draw firm conclusions about semaglutide’s effects on hard clinical endpoints such as hospitalization or mortality.

Semaglutide improves HFpEF symptoms by reducing visceral adiposity, systemic inflammation, and filling pressures, thereby enhancing exercise tolerance. These effects are primarily seen in obese patients with preserved renal and cardiac function and may not generalize to all HFpEF phenotypes. Semaglutide offers a novel therapeutic avenue for obese HFpEF patients, improving symptoms, function, and quality of life compared with existing therapies such as SGLT2 inhibitors, MRAs, and ARNIs. While cardiovascular benefits were established in T2D patients, cost-effectiveness remains a potential barrier to access and adherence, especially in lower-resource settings. Long-term trials, cost-effectiveness studies, and comparative analyses are needed to solidify its role in clinical practice, particularly across diverse HFpEF subgroups.

## Introduction and background

Heart failure with preserved ejection fraction (HFpEF) represents a growing public health challenge, characterized by a constellation of heart failure symptoms, including dyspnea, fatigue, and reduced exercise tolerance, despite a left ventricular ejection fraction (LVEF) of ≥50% [1]. Unlike its counterpart, heart failure with reduced ejection fraction (HFrEF), which benefits from well-established pharmacological interventions such as beta-blockers, angiotensin-converting enzyme inhibitors, sodium-glucose cotransporter-2 (SGLT2) inhibitors, and mineralocorticoid receptor antagonists, HFpEF remains a clinical enigma with fewer well-established treatment options, such as SGLT2 inhibitors and sacubitril/valsartan [2]. HFpEF accounts for approximately 50-60% of all heart failure cases worldwide, with an estimated 64 million individuals affected, a figure driven by an aging population and the rising prevalence of associated comorbidities including hypertension, coronary artery disease, obesity, type 2 diabetes (T2D), and chronic kidney disease (CKD) [3]. These conditions not only amplify the incidence of HFpEF but also complicate its prognosis and management, underscoring the urgent need for novel therapeutic strategies.

Historically viewed as a diagnosis of exclusion, HFpEF has evolved into a recognized syndrome with distinct pathophysiological features, including elevated left ventricular (LV) filling pressures, diastolic dysfunction, left atrial (LA) enlargement, and often elevated natriuretic peptide (NP) levels [4]. However, its heterogeneity, spanning metabolic, inflammatory, and hemodynamic phenotypes, has hindered the development of universal diagnostic criteria and effective treatments [5]. Obesity, present in over 80% of HFpEF patients, and T2D, affecting approximately 45%, are particularly prominent risk factors that exacerbate the condition through systemic inflammation, insulin resistance, and microvascular dysfunction [6,7]. Despite their prevalence, these patient subgroups have been underrepresented in clinical trials, leaving a critical gap in understanding how best to address their unique contributions to HFpEF [8]. Recent efforts have focused on more granular phenotyping through tools like the HFA-PEFF score and machine learning-based cluster analyses to facilitate individualized management. These tools offer a framework to better identify subpopulations, such as those with obesity or T2D, who may respond to targeted therapies. The therapeutic landscape for HFpEF has faced persistent challenges. Trials of agents successful in HFrEF, such as angiotensin receptor-neprilysin inhibitors (e.g., sacubitril/valsartan in PARAGON-HF) and spironolactone (TOPCAT trial), have yielded modest or inconsistent benefits in HFpEF, failing to significantly reduce mortality or comprehensively alleviate symptoms [9,10]. SGLT2 inhibitors, such as dapagliflozin and empagliflozin, have recently shown promise in reducing heart failure events across a spectrum of ejection fractions (EMPEROR-Preserved, DELIVER), yet their impact on symptom burden, exercise capacity, and quality of life in obesity- or T2D-related HFpEF remains limited [11,12]. These agents primarily provide cardiorenal and hemodynamic benefits through natriuresis and improved LV loading but have limited efficacy in weight loss and reversing systemic inflammation, which play a primary role in the pathophysiology of HFpEF.

Semaglutide, a glucagon-like peptide-1 receptor agonist (GLP-1 RA), has emerged as a compelling candidate to address these unmet needs. Initially developed for T2D management, semaglutide enhances glucose-dependent insulin secretion, suppresses glucagon release, and promotes weight loss through appetite suppression and delayed gastric emptying [13]. Its approval by the U.S. Food and Drug Administration (FDA) for chronic weight management (2.4 mg weekly subcutaneous dose) in adults with obesity or overweight with weight-related comorbidities, such as hypertension, T2D, or dyslipidemia, reflects its efficacy in achieving substantial weight reduction, with trials demonstrating losses of up to 13.3% of body weight [14]. Semaglutide, through its effects on insulin resistance, systemic inflammation, and visceral adiposity, directly targets the key pathophysiological mechanisms underpinning HFpEF, particularly in patients with obesity and T2D. Studies have shown a 43.5% reduction in C-reactive protein (CRP), and improvement in exercise capacity and quality of life in HFpEF [15,16]. HFpEF exhibits significant demographic variability, with women, who comprise the majority of patients, often presenting with greater symptom burden, more severe diastolic dysfunction, and unique cardiometabolic profiles that may influence semaglutide response. Differences in age, race, and body composition may also affect GLP-1 RA safety and efficacy, highlighting the need for more representative trials and subgroup analyses. While preliminary data are encouraging, larger long-term randomized clinical trials are needed to support these findings. Potential adverse effects such as gastrointestinal intolerance, volume depletion, and serious side effects such as pancreatitis and gallbladder disease should be considered, particularly in the elderly population. Contraindications such as a history of medullary thyroid carcinoma or multiple endocrine neoplasia syndrome type 2 also limit its use in select populations.

This review article aims to synthesize the emerging evidence on semaglutide’s application in HFpEF, focusing on its mechanisms of action, outcomes from key clinical trials, and potential to reshape clinical practice. By addressing the metabolic, inflammatory, and hemodynamic drivers of HFpEF, semaglutide offers a novel therapeutic avenue, potentially bridging the gap between patient-centered outcomes (e.g., symptom relief, improved quality of life) and cardiovascular risk reduction. The following sections will explore the background of HFpEF, semaglutide’s pharmacological profile, and the clinical data supporting its use, culminating in a discussion of its implications and future directions.

Epidemiology and burden of HFpEF 

The prevalence of HFpEF has grown substantially over recent decades, reflecting demographic shifts, rising life expectancy, and increasing cardiometabolic risk. Current estimates indicate that HFpEF represents half or more of all HF cases globally, and this proportion is expected to rise, particularly in regions where obesity rates are projected to approach 50% by 2030 [17,18]. HFpEF disproportionately affects older adults, women, and individuals with obesity or metabolic disease, populations that present with distinct clinical profiles and may respond differently to therapies [19]. Unlike heart failure with reduced ejection fraction, where survival has improved with guideline-directed medical therapy, HFpEF patients face a persistently high mortality risk, with 5-year survival rates hovering around 50% post-hospitalization [20]. This disparity reflects both the complexity of HFpEF and the paucity of effective interventions.

Subgroup analyses from large trials (e.g., PARAGON-HF, TOPCAT) have provided insight into potential responders to therapies like sacubitril/valsartan and spironolactone, but overall effects on mortality or sustained symptom relief have been modest [9,10]. More recently, SGLT2 inhibitors such as dapagliflozin (DELIVER) and empagliflozin (EMPEROR-Preserved) have shown reductions in HF hospitalizations across the LVEF spectrum [11]. However, their efficacy in symptom relief, exercise capacity, and metabolic modulation in patients with obesity-related HFpEF remains constrained. These limitations underscore the need for phenotype-specific therapeutic strategies that go beyond hemodynamic control.

**Figure 1:**
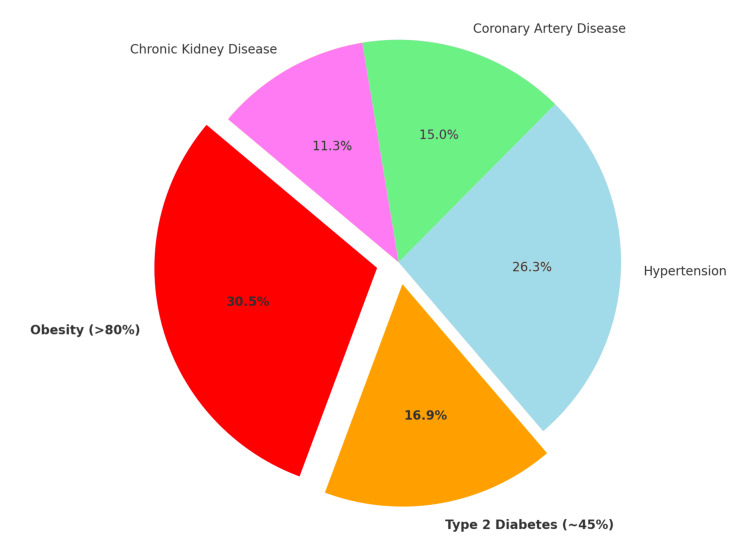
Prevalence of comorbidities in heart failure with preserved ejection fraction High prevalence of obesity (>80%) and type 2 diabetes (T2D, ~45%) in HFpEF patients, alongside other common comorbidities such as hypertension, coronary artery disease (CAD), and chronic kidney disease (CKD). Figure created by authors using the data from refs [3,6,7].

The pie chart in Figure 1 illustrates the high prevalence of obesity (>80%) and T2D (~45%) in HFpEF patients, alongside other common comorbidities such as hypertension, coronary artery disease (CAD), and CKD. Percentages reflect prevalence with potential overlap, as patients may have multiple comorbidities [3,6,7].

Pathophysiology and heterogeneity 

Heart failure with preserved ejection fraction (HFpEF) is not a single disease, but a complex condition involving multiple underlying causes and a wide range of clinical features. Core features include impaired left ventricular relaxation and increased stiffness, leading to elevated filling pressures during diastole, particularly under stress (e.g., exercise) [21]. These abnormalities are often accompanied by left ventricular hypertrophy, left atrial dilation, and subtle systolic dysfunction despite preserved ejection fraction, as well as extracardiac manifestations such as pulmonary hypertension and skeletal muscle impairment [22]. Elevated NPs, such as B-type NP (BNP), are common but not universal, complicating diagnosis in obese patients where NP production and clearance are altered [23]. The understanding of HFpEF has evolved significantly over the past decade. Early definitions relied heavily on ejection fraction as a distinguishing feature from HFrEF, an oversimplification that ignored underlying mechanisms [24]. Contemporary frameworks, informed by studies like those of Shah et al., emphasize phenotyping based on clinical, imaging, laboratory, and molecular data [25]. For instance, Fayol et al. classified HFpEF into three etiologic subgroups: idiopathic (dominated by non-cardiac comorbidities), secondary (linked to myocardial hemodynamic abnormalities), and metabolic (characterized by obesity, T2D, and severe diastolic dysfunction), with prognosis worsening across these categories [26]. The metabolic phenotype, marked by a fourfold increased mortality risk, underscores the pivotal role of obesity and T2D in driving adverse outcomes [25].

Obesity contributes to HFpEF through multiple pathways. Excess visceral adipose tissue promotes a chronic inflammatory state, with macrophage infiltration releasing cytokines (e.g., interleukin-6, tumor necrosis factor-alpha) that impair endothelial nitric oxide bioavailability and increase myocardial stiffness via reduced cyclic GMP and protein kinase G activity [27]. This inflammatory cascade exacerbates coronary microvascular dysfunction, a key feature of HFpEF, and elevates plasma volume and cardiac workload [28]. T2D amplifies these effects through hyperglycemia, lipotoxicity, and hyperinsulinemia, which foster advanced glycation end-products, concentric left ventricular remodeling, and diabetic cardiomyopathy, a restrictive phenotype prevalent in HFpEF [29]. The interplay of these factors results in a vicious cycle of hemodynamic stress, exercise intolerance, and symptom exacerbation, particularly in obese diabetic patients [30].

**Figure 2:**
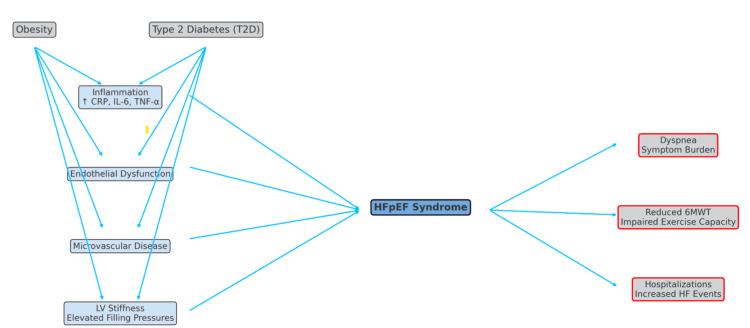
Pathophysiological mechanisms in obese HFpEF HFpEF: heart failure with preserved ejection fraction.

The flowchart in Figure 2 illustrates the cascade from obesity and T2D to HFpEF syndrome through inflammation, endothelial dysfunction, microvascular disease, and left ventricular (LV) stiffness, resulting in clinical outcomes such as dyspnea, reduced six-minute walk test (6MWT) distance, and increased hospitalizations. Light blue arrows indicate detrimental pathways.

Clinical challenges and therapeutic gaps 

Diagnosing HFpEF remains challenging due to its heterogeneity and overlap with other conditions (e.g., pulmonary disease, obesity-related dyspnea). The H2FPEF score and updated ESC guidelines have improved diagnostic precision by integrating clinical and echocardiographic parameters, yet many patients remain undiagnosed until advanced stages [1,31]. Therapeutically, HFpEF has proven resistant to strategies effective in HFrEF. The PARAGON-HF trial of sacubitril/valsartan showed a modest reduction in heart failure hospitalizations but no significant mortality benefit [9]. Spironolactone, evaluated in TOPCAT, improved cardiac structure but failed to reduce overall mortality, with benefits limited to specific subgroups [10]. Beta-blockers and angiotensin-converting enzyme (ACE) inhibitors, cornerstones of HFrEF management, lack consistent efficacy in HFpEF, reflecting differences in underlying pathophysiology [32]. SGLT2 inhibitors have recently offered hope, with the DELIVER and EMPEROR-Preserved trials demonstrating reductions in composite endpoints of worsening heart failure or cardiovascular death in patients with LVEF >40% [11,12]. However, their effects on symptom burden and obesity-specific outcomes are less pronounced, and their mechanism, primarily natriuretic and decongestive, does not fully address the metabolic and inflammatory drivers of HFpEF [33]. Weight loss, a proven benefit in obese HFpEF patients, improves exercise capacity and quality of life, as shown in trials of caloric restriction and exercise, yet achieving and sustaining it through lifestyle alone is challenging [34]. This gap has fueled interest in pharmacological agents like semaglutide, which combine weight reduction with metabolic and cardiovascular benefits.

**Figure 3:**
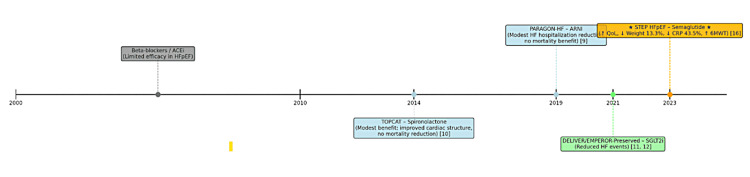
Evolution of HFpEF therapies and semaglutide's emergence Figure created by authors using data from refs [9-12,16,32]. HFpEF: heart failure with preserved ejection fraction.

Figure 3 shows the timeline tracking major therapeutic milestones in HFpEF management, from early therapies (beta-blockers, angiotensin-converting enzyme inhibitors (ACEi)) to recent advances (sodium-glucose cotransporter-2 inhibitors (SGLT2i), semaglutide). Outcomes reflect key findings: limited efficacy for beta-blockers/ACEi [32], modest benefits for spironolactone (TOPCAT, 2014) [10], neutral results for sacubitril/valsartan (PARAGON-HF, 2019) [9], reduced heart failure (HF) events for SGLT2i (DELIVER/EMPEROR-Preserved, 2021-2022) [11,12], and improved quality of life (QoL), weight loss, C-reactive protein (CRP) reduction, and 6-minute walk test (6MWT) distance with semaglutide (STEP HFpEF, 2023) [16].

Rationale for semaglutide in HFpEF 

Semaglutide’s potential in HFpEF stems from its ability to target the syndrome’s metabolic and inflammatory roots. Approved for T2D (0.25-1 mg weekly subcutaneous or 3-14 mg daily oral) and obesity (2.4 mg weekly), it induces significant weight loss (e.g., 6-13.3% in STEP trials), improves glycemic control, and reduces cardiovascular events (e.g., stroke in SUSTAIN 6) without hypoglycemia risk [13-15]. Its anti-inflammatory effects, evidenced by a 43.5% CRP reduction in HFpEF trials, address endothelial dysfunction and myocardial stiffness, while reduced diuretic needs suggest decongestive potential [16,35]. These properties align with HFpEF’s obesity-driven phenotype, where visceral fat, inflammation, and insulin resistance predominate, and offer a counterbalance to the limited efficacy of traditional therapies. Moreover, patient priorities, symptom relief, and functional improvement may be better met by semaglutide’s broad actions, as formal interviews indicate these clinical outcomes rival survival in importance [36]. This background sets the stage for a detailed exploration of semaglutide’s mechanisms and clinical evidence in HFpEF. 

## Review

Mechanism of action

Semaglutide, a glucagon-like peptide-1 receptor agonist (GLP-1 RA), exerts a multifaceted mechanism of action that aligns with the metabolic, inflammatory, and hemodynamic challenges of heart failure with preserved ejection fraction (HFpEF), particularly in patients with obesity and T2D [37]. Designed for prolonged activity, semaglutide incorporates structural modifications to resist degradation and allow once-weekly subcutaneous dosing or daily oral formulations, improving patient adherence and long-term tolerability [38-40].

Metabolic effects

Semaglutide’s primary metabolic actions stem from its agonism of GLP-1 receptors, widely expressed in pancreatic β-cells, the gastrointestinal tract, and the central nervous system. It enhances glucose-dependent insulin secretion, suppresses glucagon release during hyperglycemia, and inhibits hepatic gluconeogenesis, collectively lowering blood glucose and HbA1c without risking hypoglycemia, a critical advantage over traditional antidiabetic agents in HFpEF patients prone to comorbidities [41,42]. In T2D, these effects mitigate hyperglycemia-induced lipotoxicity and advanced glycation end-products, which contribute to microvascular dysfunction and myocardial stiffness in HFpEF [29]. Clinical trials, such as SUSTAIN, report HbA1c reductions of 1.5-1.8% with semaglutide, underscoring its potency [15].

Weight loss and appetite regulation

Beyond glycemic control, semaglutide induces significant weight loss, a pivotal benefit for obese HFpEF patients. It delays gastric emptying, enhancing satiety, and acts on hypothalamic and brainstem appetite centers via vagal nerve pathways and direct central nervous system effects, reducing energy intake [43]. The Semaglutide Treatment Effect in People with Obesity (STEP) trials demonstrated average weight reductions of 6% by week 12 and 12-13.3% by weeks 28-52 with a 2.4 mg weekly dose, far exceeding outcomes with lifestyle interventions alone [14].

Anti-inflammatory and cardiovascular effects

Semaglutide’s anti-inflammatory properties further enhance its relevance to HFpEF. Obesity and T2D foster a pro-inflammatory state, with adipose tissue macrophages releasing cytokines (e.g., IL-6, TNF-α) that impair endothelial nitric oxide (NO) bioavailability, reduce cyclic GMP and protein kinase G activity, and increase myocardial stiffness [27]. In the STEP HFpEF trials, semaglutide reduced C-reactive protein (CRP) by 43.5% compared to 7.3% with placebo (P < 0.001) [16]. This level of CRP reduction is comparable to anti-inflammatory interventions associated with improved cardiovascular outcomes and may reflect a meaningful attenuation of systemic and myocardial inflammation. Such reductions may also improve endothelial function, coronary microvascular perfusion, and interstitial remodeling, core pathophysiologic mechanisms in HFpEF. While semaglutide demonstrated cardiovascular benefits in SUSTAIN 6 by reducing stroke risk, PIONEER 6 showed a non-significant trend toward reduced cardiovascular death [15,27]. Whether these benefits are unique to semaglutide or reflect a broader GLP-1 RA class effect remains under investigation, although head-to-head data suggest comparable metabolic effects across agents, with variability in cardiovascular endpoints.

Hemodynamic and decongestive potential

Emerging evidence suggests that semaglutide may influence fluid dynamics in HFpEF. The STEP HFpEF analysis presented at Heart Failure 2024 reported a reduced need for loop diuretics, a lower average diuretic dose, and a higher likelihood of diuretic de-escalation compared to placebo [35]. While the precise mechanism remains speculative, it is possibly related to weight loss, reducing plasma volume, improved renal hemodynamics, and potential neurohormonal modulation. This finding positions semaglutide as a potential adjunct to traditional decongestive strategies, addressing a critical aspect of HFpEF management [34].

**Figure 4:**
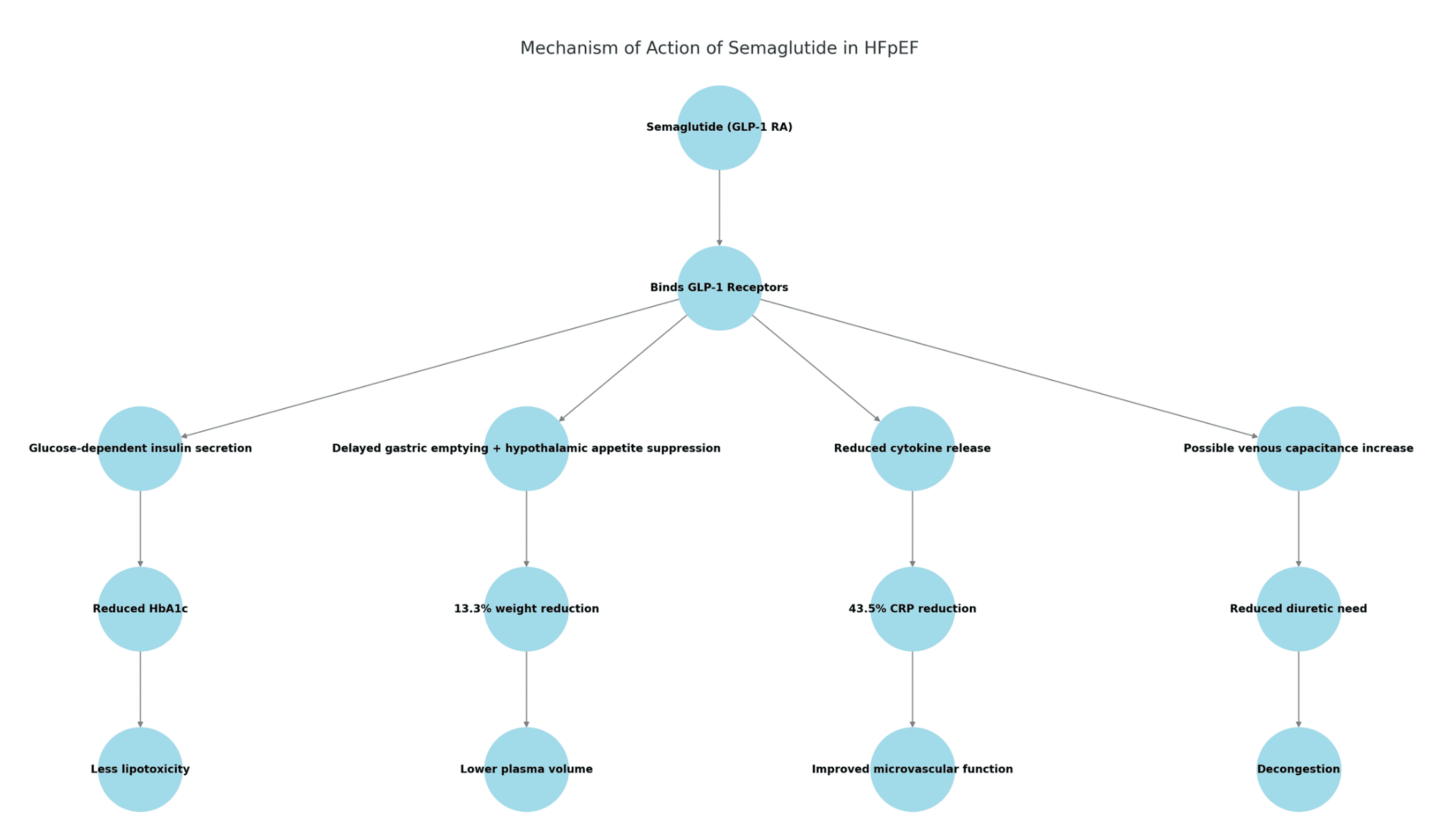
Mechanism of action of semaglutide in HFpEF HFpEF: heart failure with preserved ejection fraction.

In summary, semaglutide’s mechanisms, including metabolic optimization, weight reduction, anti-inflammatory action, and possible decongestive effects, directly target the obesity-driven, inflammatory, and hemodynamic hallmarks of HFpEF (Figure 4). These properties distinguish it from existing therapies like SGLT2 inhibitors, which primarily address fluid overload, and provide a rationale for its evaluation in this population. 

Key clinical trials

The clinical evidence supporting semaglutide in heart failure with preserved ejection fraction (HFpEF) and related conditions derives from a series of robust trials, ranging from cardiovascular outcome studies in T2D to targeted investigations in HFpEF with obesity and T2D. Below, we review the pivotal trials, SUSTAIN 6, PIONEER 6, STEP HFpEF, STEP HFpEF DM, JACC Study, and SUMMIT, focusing on their design, outcomes, and implications for HFpEF.

SUSTAIN 6: semaglutide and cardiovascular outcomes in T2D

The SUSTAIN 6 trial enrolled 3,297 patients with T2D and high cardiovascular risk (83% with established cardiovascular or kidney disease), randomizing them to semaglutide (0.5 or 1.0 mg weekly) or placebo for 104 weeks [15]. The primary endpoint, which is the first occurrence of cardiovascular death, nonfatal myocardial infarction (MI), or nonfatal stroke, demonstrated semaglutide’s non-inferiority and superiority. Nonfatal stroke occurred in 1.6% of the semaglutide group vs. 2.7% of placebo (HR 0.61, 95% CI 0.38-0.99, P = 0.04), while nonfatal MI showed a trend toward reduction (2.9% vs. 3.9%, HR 0.74, 95% CI 0.51-1.08, P = 0.12). Cardiovascular death rates were similar (2.7% vs. 2.8%). Gastrointestinal side effects (e.g., nausea, vomiting) were more common with semaglutide, but overall safety was favorable [15]. While not HFpEF-specific, these findings established semaglutide’s cardiovascular benefits, laying the groundwork for its exploration in heart failure populations with overlapping risk profiles.

PIONEER 6: oral semaglutide and cardiovascular outcomes in T2D

PIONEER 6 assessed oral semaglutide in 3,183 patients with T2D and high cardiovascular risk over a median of 15.9 months [27]. Participants received oral semaglutide (up to 14 mg daily) or placebo, with the primary endpoint being major adverse cardiovascular events (MACE): cardiovascular death, nonfatal MI, or nonfatal stroke. MACE rates were 3.8% with semaglutide vs. 4.8% with placebo (HR 0.79, 95% CI 0.57-1.11, P < 0.001 for non-inferiority), with significant reductions in cardiovascular death (0.9% vs. 1.9%, HR 0.49, 95% CI 0.27-0.92) and all-cause mortality (1.4% vs. 2.8%, HR 0.51, 95% CI 0.31-0.84). The trial confirmed oral semaglutide’s safety and efficacy, broadening its applicability, though HFpEF-specific outcomes were not evaluated [27]. These results reinforce semaglutide’s potential to mitigate cardiovascular risk factors prevalent in HFpEF, such as atherosclerosis and metabolic dysfunction.

STEP HFpEF: semaglutide in obesity-related HFpEF

The STEP HFpEF trial was a landmark double-blind, randomized study of 529 patients with HFpEF (EF ≥45%) and obesity (BMI ≥30), assigned to semaglutide 2.4 mg weekly or placebo for 52 weeks [16]. The primary endpoint, that is, the change in the Kansas City Cardiomyopathy Questionnaire Clinical Summary Score (KCCQ-CSS), improved significantly with semaglutide (+16.6 points) vs. placebo (+8.7 points), with a between-group difference of 7.8 points (P < 0.001), exceeding the 5-point threshold for a clinically meaningful improvement in symptoms and quality of life. Secondary endpoints included weight reduction (−13.3% vs. −2.6%, P < 0.001), increased 6-minute walk test (6MWT) distance (+21.5 meters vs. +1.2 meters, P < 0.001), and a 43.5% vs. 7.3% reduction in CRP (P < 0.001). Serious adverse events were less frequent with semaglutide (13.3% vs. 26.7%), though gastrointestinal side effects led to discontinuation in some cases [16]. Semaglutide’s impact on inflammation and weight reduction may address central HFpEF pathophysiologic mechanisms such as myocardial stiffness, vascular dysfunction, and exercise intolerance. CRP reduction suggests attenuation of systemic inflammation, a known contributor to HFpEF progression. These mechanistic effects align with the observed clinical benefits in symptoms and functional capacity, helping to close a critical therapeutic gap in obesity-related HFpEF, as shown in Figure 2.

STEP HFpEF DM: semaglutide in HFpEF with T2D

The STEP HFpEF DM trial extended these findings to 616 patients with HFpEF (EF ≥45%), obesity (BMI ≥30), and T2D, randomized to semaglutide 2.4 mg weekly or placebo for 52 weeks across 108 sites [29]. The KCCQ-CSS increased by 13.7 points with semaglutide vs. 6.4 points with placebo (difference 7.3, P < 0.001), with weight loss of 9.8% vs. 3.4% (P < 0.001) and 6MWT gains of 14.3 meters (P = 0.008). CRP reduction mirrored STEP HFpEF (43.5%, P < 0.001), and serious adverse events were lower (17.7% vs. 28.8%). The hierarchical composite endpoint (death, HF events, KCCQ-CSS, and 6MWT) favored semaglutide (win ratio 1.58, P < 0.001), reinforcing its efficacy in this dual-comorbidity cohort [29]. The slightly lower weight loss compared to STEP HFpEF may reflect T2D-related metabolic differences, but the benefits across symptom, function, and inflammation domains remained robust.

**Figure 5:**
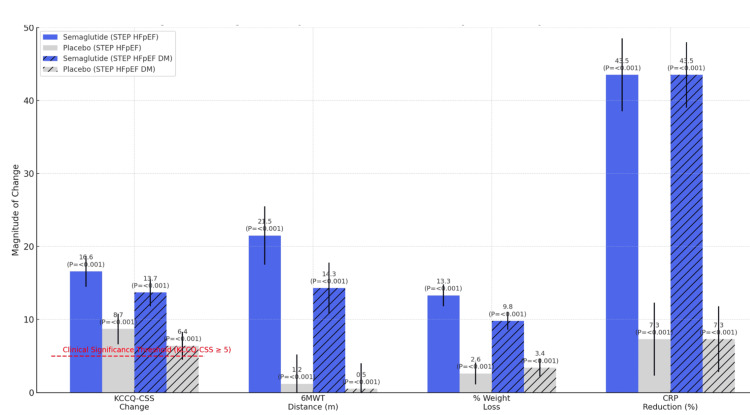
Semaglutide outperforms placebo in STEP HFpEF trials Bar chart created by the authors using data from refs [16,29]. HFpEF: heart failure with preserved ejection fraction, STEP: Semaglutide Treatment Effect in People With Obesity.

Figure 5 shows the bar graph comparing semaglutide vs. placebo effects on Kansas City Cardiomyopathy Questionnaire Clinical Summary Score (KCCQ-CSS, points), weight loss (%), 6-minute walk test (6MWT) distance (meters), and C-reactive protein (CRP) reduction (%) in the STEP HFpEF (n = 529) and STEP HFpEF DM (n = 616) trials. Error bars represent standard deviation. P-values indicate statistical significance. Dashed line at 5 points denotes the clinical significance threshold for KCCQ-CSS [16,29].

Table 1 compares the key aspects and outcomes of the STEP-HFpEF and STEP-HFpEF DM trials, which evaluated the effects of semaglutide 2.4 mg once weekly in obese patients with heart failure with preserved ejection fraction (HFpEF), with and without T2D.

**Table 1:** A summary of STEP-HFpEF and STEP-HFpEF DM trials Table created by the authors using data from refs [16,29]. HFpEF: heart failure with preserved ejection fraction, STEP: Semaglutide Treatment Effect in People With Obesity.

Aspect	STEP-HFpEF	STEP-HFpEF DM
Population type	Obese patients with HFpEF (without type 2 diabetes)	Obese patients with HFpEF and type 2 diabetes
Dose	Semaglutide 2.4 mg once weekly	Semaglutide 2.4 mg once weekly
Endpoints	Symptom improvement: Kansas City Cardiomyopathy Questionnaire Symptom Improvement (KCCQ-CSS Clinical Summary Score	Symptom improvement: KCCQ-CSS Clinical Summary Score
	Weight loss	Weight loss
	Inflammatory Markers: C-reactive protein (CRP) reduction	Inflammatory markers: C-reactive protein (CRP) reduction
Clinical effects	Symptom improvement: +16.6 points in KCCQ-CSS	Symptom improvement: +13.7 points in KCCQ- Effects CSS
	Functional gains: +21.5 meters in 6MWT	Functional gains: +12.7 meters in 6MWT
	Weight loss: -13.3% body weight	Weight loss: -9.8% body weight
	CRP reduction: -43.5%	CRP reduction: -43.5%
	Safety/adverse effects: well-tolerated with no significant safety concerns	Safety/adverse effects: well-tolerated with no significant safety concerns

JACC study: GLP-1 RA with SGLT2 inhibitors in HFpEF

A retrospective cohort study analyzed 29,922 patients with T2D and obesity-related HFpEF on SGLT2 inhibitors, comparing 7,044 patients who received additional GLP-1 RA therapy (including semaglutide) with 7,044 on SGLT2 inhibitors alone over 12 months [30]. Combination therapy reduced HF exacerbations (HR 0.62, 95% CI 0.58-0.67), all-cause mortality (HR 0.64, 95% CI 0.54-0.75), and hospitalizations (HR 0.74, 95% CI 0.71-0.78), alongside lower risks of atrial fibrillation, pulmonary hypertension, and renal injury. Importantly, the combination was associated with an elevated risk of diabetic retinopathy, an established complication of rapid glycemic improvement with GLP-1 RAs. This underscores the need for baseline retinal screening and gradual glycemic control [30]. While not exclusively semaglutide-focused, this study suggests synergistic benefits with SGLT2 inhibitors, a class already established in HFpEF-enhancing the case for combination strategies.

SUMMIT trial: tirzepatide as a comparator

The SUMMIT trial, while not semaglutide-specific, provides context by evaluating tirzepatide (a dual GLP-1/GIP agonist) in 731 obese HFpEF patients (EF ≥50%) over 104 weeks [31]. Tirzepatide (up to 15 mg weekly) reduced cardiovascular death or worsening HF (9.9% vs. 15.3%, HR 0.62, P = 0.026) and improved KCCQ-CSS (19.5 vs. 12.7 points, P < 0.001) compared to placebo, though gastrointestinal discontinuation rates were higher (6.3% vs. 1.4%) [31]. These findings parallel semaglutide’s benefits, suggesting a class effect of incretin-based therapies in HFpEF, with tirzepatide’s dual agonism potentially offering incremental advantages.

Across these trials, KCCQ-CSS improvements and hazard ratios for cardiovascular events provide a comparative lens on therapeutic efficacy. Tirzepatide demonstrated the greatest symptomatic improvement (KCCQ-CSS gain of 6.8 points over placebo), while the GLP-1 RA + SGLT2i combination yielded strong prognostic benefits, including a 36% relative risk reduction in heart failure events. These findings suggest both additive and potentially complementary effects of metabolic therapies in HFpEF. Of note, EF inclusion thresholds varied (≥45% in STEP and JACC studies vs. ≥50% in SUMMIT), which may influence generalizability. Patients with EF between 45-50%, often excluded from stricter HFpEF trials, represent a transitional group that may benefit similarly, though further stratified analyses are needed.

Table 2 provides a comparative summary of these trials, synthesizing their designs, outcomes, and limitations.

**Table 2:** Comparative summary of key clinical trials evaluating semaglutide and related therapies in HFpEF and related conditions Table created by the authors using the data from refs [15,16,27,29-31]. T2D: type 2 diabetes, CV: cardiovascular, MI: myocardial infarction, MACE: major adverse cardiovascular events, HFpEF: heart failure with preserved ejection fraction, EF: ejection fraction, BMI: body mass index, KCCQ-CSS: Kansas City Cardiomyopathy Questionnaire Clinical Summary Score, CRP: C-reactive protein, GLP-1 RA: glucagon-like peptide-1 receptor agonist, SGLT2: sodium-glucose cotransporter 2, GIP: glucose-dependent insulinotropic polypeptide, GI: gastrointestinal.

Study	Population	Intervention	Primary Endpoint	Key Findings	Limitations	References
SUSTAIN 6	3,297 T2D patients with high CV risk (83% with established CV or kidney disease)	Semaglutide 0.5–1.0 mg weekly vs. placebo	CV death, nonfatal MI, nonfatal stroke (MACE)	- Reduced nonfatal stroke (HR 0.61, P=0.04) - Trend toward reduction in nonfatal MI (HR 0.74, P=0.12) - No effect on CV death	- Not HFpEF-specific - Short follow-up (104 weeks) - Focused on CV events, not HFpEF-specific endpoints	Marso et al. (2016) [15].
PIONEER 6	3,183 T2D patients with high CV risk	Oral semaglutide up to 14 mg daily vs. placebo	Major adverse CV events (MACE: CV death, nonfatal MI, nonfatal stroke)	- Non-inferiority for MACE (HR 0.79, P<0.001) - Significant reduction in CV death (HR 0.49, P=0.01) and all-cause mortality (HR 0.51, P<0.001)	- No HFpEF-specific outcomes - Short follow-up (~16 months) - Focused on MACE, not HFpEF-specific symptoms or function	Husain et al. (2019) [27]
STEP HFpEF	529 obese HFpEF patients (EF ≥45%), BMI ≥30	Semaglutide 2.4 mg weekly vs. placebo	Change in Kansas City Cardiomyopathy Questionnaire Clinical Summary Score (KCCQ- CSS)	- Improved KCCQ-CSS (+16.6 vs. +8.7, P<0.001) - 13.3% weight loss vs. 2.6% with placebo (P<0.001) - 43.5% reduction in CRP (P<0.001)	- No data on hard endpoints (e.g., HF hospitalizations) - 1- year follow-up - Focused on obese patients, limiting applicability to non-obese HFpEF	Kosiborod et al. (2023) [16]
STEP HFpEF DM	616 obese HFpEF patients with T2D (EF ≥45%), BMI ≥30	Semaglutide 2.4 mg weekly vs. placebo	Change in KCCQ- CSS	- KCCQ-CSS increase of 13.7 points vs. 6.4 with placebo (P<0.001) - 9.8% weight loss vs. 3.4% with placebo (P<0.001) - 43.5% CRP reduction (P<0.001)	- No assessment of long-term outcomes (e.g., HF hospitalizations) - Focus on patients with T2D, differing from non-diabetic HFpEF - 1- year follow-up	Kosiborod et al, (2024) [29]
JACC Study	29,922 T2D, obese HFpEF patients on SGL2 inhibitors (7,044 with GLP-1 RA vs. 7,044 without)	GLP-1 RA (including semaglutide) + SGLT2 inhibitors vs. SGLT2 inhibitors alone	HF exacerbations, all-cause mortality, hospitalizations	- Reduced HF exacerbations (HR 0.62), hospitalizations (HR 0.74), and all-cause mortality (HR 0.64)	- Retrospective study - Increased risk of diabetic retinopathy with combination therapy	Patel et al, (2024) [30]
SUMMIT Trial	731 obese HFpEF patients (EF ≥50%)	Tirzepatide (GLP-1/GIP agonist) up to 15 mg weekly vs. placebo	CV death or worsening HF	- Reduced CV death or worsening HF (HR 0.62, P=0.026) - Significant improvement in KCCQ-CSS (+19.5 vs. +12.7, P<0.001)	- Focused on tirzepatide, not semaglutide - Higher GI discontinuation rates with tirzepatide	Packer et al, (2024) [31]

Discussion and clinical implications

Heart failure with preserved ejection fraction (HFpEF) poses a significant clinical burden, particularly in patients with obesity, who often experience more severe symptoms, reduced functional capacity, and poorer quality of life [2,19,20,22]. Symptom relief and improved physical function are increasingly recognized as therapeutic priorities, on par with reducing mortality and hospitalizations [36]. However, conventional heart failure therapies have shown limited efficacy in achieving these goals in HFpEF. Trials such as TOPCAT, Aldo-DHF, and PARAGON-HF failed to demonstrate significant reductions in mortality, despite modest improvements in hospitalization rates and cardiac structure [9,10]. Obesity-related HFpEF is characterized by distinct hemodynamic and inflammatory features, including volume overload, impaired NP signaling, and systemic inflammation [22,23]. These factors complicate management and contribute to worse outcomes. While lifestyle interventions can improve functional status, pharmacologic options targeting this phenotype have been limited until recently [34].

Emerging therapies such as semaglutide, a GLP-1 receptor agonist, offer a novel, multifaceted approach. Beyond weight loss, semaglutide improves symptoms, exercise capacity, and inflammatory markers, addressing several key pathophysiologic mechanisms in obese HFpEF [16,29]. In the STEP HFpEF and STEP HFpEF DM trials, semaglutide produced clinically meaningful increases in the Kansas City Cardiomyopathy Questionnaire Clinical Summary Score (KCCQ-CSS), reduced C-reactive protein (CRP) by over 40%, and improved 6-minute walk distance. These effects are particularly notable given that even guideline-directed therapies such as SGLT2 inhibitors, spironolactone, or ARNI (e.g., sacubitril/valsartan) have shown more modest improvements in patient-reported outcomes [16,29].

Semaglutide’s ability to target obesity, inflammation (via CRP), and metabolic dysfunction aligns well with a precision medicine approach, wherein therapy is tailored to distinct phenotypes within HFpEF. This precision approach is supported by identifiable clinical features such as elevated BMI, elevated inflammatory biomarkers (e.g., CRP), insulin resistance or T2D, and exercise intolerance. These parameters may help clinicians select patients most likely to benefit from semaglutide, and ongoing studies may refine biomarker-based treatment algorithms.

**Figure 6:**
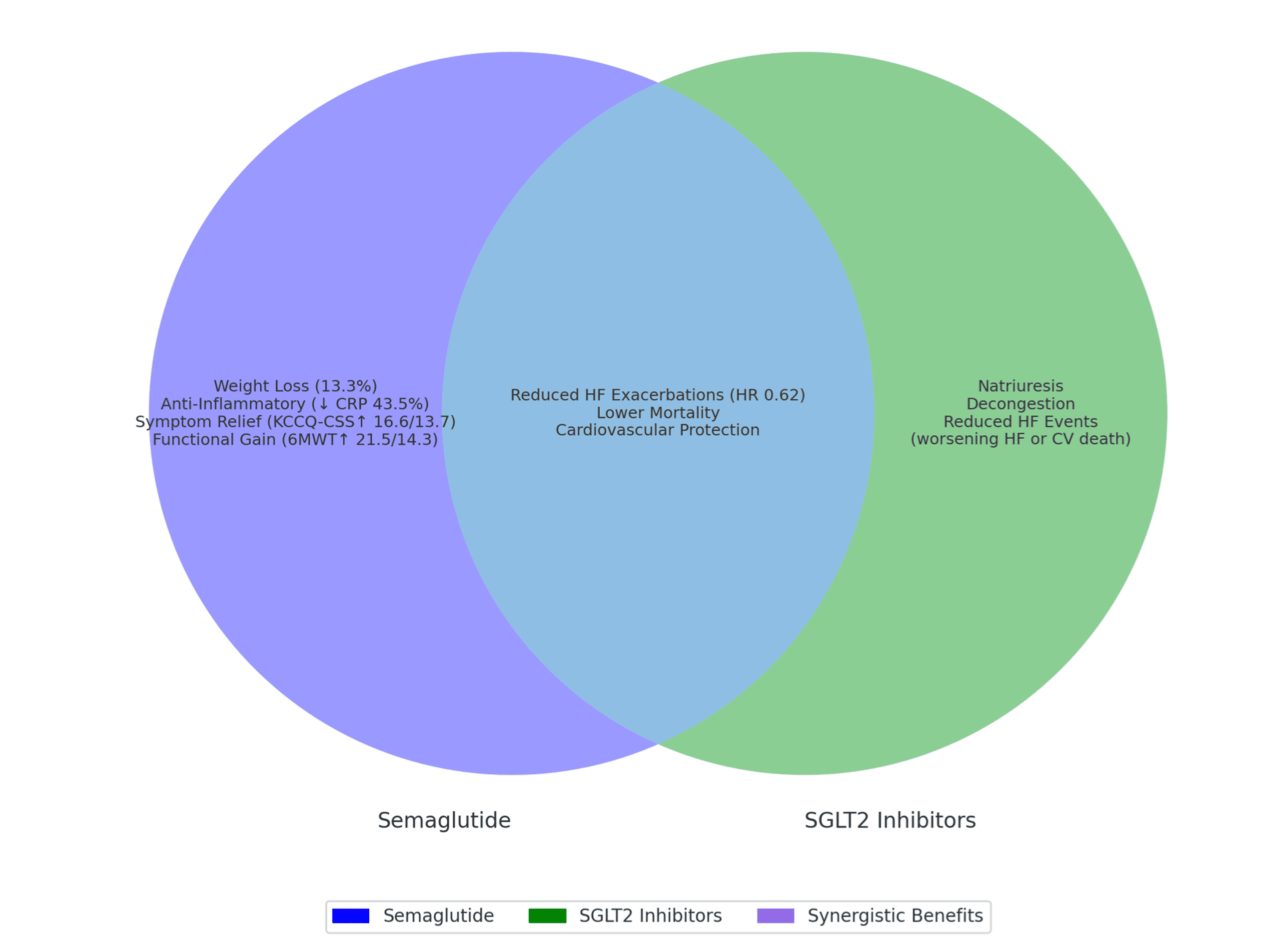
Synergetic effects of semaglutide and SGL2 inhibitors Image created by the authors using the data from ref [31].

Semaglutide’s benefits complement those of SGLT2 inhibitors, suggesting a potential synergistic strategy that targets both congestion and metabolic dysfunction [30]. However, the study also noted an increased risk of diabetic retinopathy, a complication associated with rapid glucose lowering. As a result, clinicians should consider retinal screening prior to initiation of combination therapy. Compared to agents like tirzepatide, which showed cardiovascular benefits but higher rates of gastrointestinal side effects, semaglutide appears particularly effective in improving physical performance, an outcome highly valued by patients (Figure 6) [31].

Despite the promising efficacy profile, real-world implementation of semaglutide remains challenging. Barriers include high cost, limited insurance coverage, and prescribing logistics, particularly in primary care settings. While semaglutide has been approved for obesity and diabetes, its role in HFpEF is still evolving and has not yet been incorporated into major heart failure guidelines. Reimbursement pathways often lag behind evidence, and disparities in access, especially among underserved populations, may widen existing healthcare gaps. Moreover, adherence may be compromised by gastrointestinal side effects, weekly injection burden, and out-of-pocket costs.

Trial limitations must also be acknowledged. In the STEP HFpEF trials, dropout rates due to adverse events, mainly gastrointestinal, were higher in the semaglutide arms. Additionally, while the trials were double-blind and placebo-controlled, the visible weight loss in treated patients may have compromised blinding, potentially influencing subjective endpoints such as KCCQ scores. Long-term data beyond 52 weeks, including on heart failure hospitalizations and mortality, are still awaited [35,40].

Limitations of STEP HFpEF trials

The STEP HFpEF trials, while groundbreaking, have several limitations that temper their interpretation and generalizability. First, their primary design focused on symptoms, physical limitations, and exercise function (KCCQ-CSS, 6MWT, weight loss), not clinical events such as heart failure hospitalizations or urgent visits. Second, the one-year follow-up duration restricts conclusions about long-term efficacy and safety. While semaglutide showed sustained improvements in KCCQ-CSS, 6MWT, and weight loss compared to placebo, the durability of these effects beyond 52 weeks remains unproven. Third, generalizability is constrained by the trials’ exclusive focus on obese HFpEF patients (BMI ≥30). This limits applicability to non-obese HFpEF phenotypes, e.g., those driven by hypertension or aging without significant adiposity, which may differ in pathophysiology and treatment response. Exclusion of non-diabetic patients in STEP HFpEF-DM further narrows relevance, as T2D alters metabolic and inflammatory profiles, potentially amplifying semaglutide’s effects. This phenotypic specificity reduces external validity, particularly for leaner or metabolically healthy HFpEF cohorts. These limitations highlight the need for larger, long-term studies to more comprehensively define the role of semaglutide across the spectrum of heart failure.

## Conclusions

STEP-HFpEF and STEP HFpEF-DM demonstrated semaglutide’s potential in improving symptoms, exercise capacity, inflammation, and weight among obese HFpEF patients-with or without T2D-while maintaining a favorable safety profile. While reduction in hospitalization was not assessed in the STEP trials, observational data suggest possible benefits in healthcare utilization. Additive effects with SGLT2 inhibitors are inferred from real-world retrospective data, though not yet confirmed in randomized trials. Semaglutide offers a promising metabolic approach for obesity-related HFpEF by improving patient-reported outcomes (KCCQ score, physical function), which are increasingly prioritized in HFpEF management. Future research should focus on long-term outcomes, head-to-head comparisons with other agents (e.g., tirzepatide, SGLT2is), and trials in the non-obese HFpEF population. Monitoring known risks such as gastrointestinal intolerance and diabetic retinopathy is essential, particularly in high-risk populations, to ensure safe implementation of semaglutide in heart failure management.
